# Difference in Using Protrusion Face Mask before or after Rapid Palatal Expansion in Skeletal Class III Children: A Preliminary Study

**DOI:** 10.3390/children9101535

**Published:** 2022-10-08

**Authors:** Patrizia Lucchi, Marco Rosa, Giovanni Bruno, Alberto De Stefani, Francesca Zalunardo, Antonio Gracco

**Affiliations:** 1 Department of Neuroscience, University of Padova, 35122 Padua, Italy; 2 Department of Pharmacological Sciences, University of Padua, 35100 Padua, Italy

**Keywords:** skeletal class III, rapid palatal expansion, palatal expansion, early treatment

## Abstract

Treatment of third-class malocclusions often presents a challenge for orthodontists. Skeletal disharmony is often associated with dental malposition. There are several therapeutic choices, including the use in combination of transverse expansion of the maxilla with rapid palatal expander (RPE) and posterior-anterior traction with a Delaire face mask (FM). The purpose of the study is to verify whether there are significant differences in the treatment outcome in the case of use of a face mask followed by a palatal expander or with the sequence of these auxiliaries reversed. **Subject and Methods:** The two groups were both made up of 13 patients, subdivided into group A, i.e., those whose sequence involved the use of extraoral traction first and then the disjunctor, and those with an inverted sequence in group B. Some cephalometric parameters and dento-skeletal characteristics were evaluated pre-treatment (t0) and at the end of therapy (t1). **Results:** Considering the T1–T0 of group A (Delaire + rapid palatal expander), the evaluation of the results obtained in this work allows us to observe how within group A there is a significant improvement in the Witts and Nanda indices and facial convexity. Group B (treated with the palate disjunctor sequence followed by traction with Delaire’s mask) showed a significant improvement in ANB, in AoBo, and AppBpp values and in convexity. The two groups were comparable, and no statistically significant difference was highlighted. **Discussion:** The early therapy of the third skeletal classes by means of a rapid palate expander and face mask is effective. There is no statistically significant difference in the two groups who performed the therapy in reverse mode. This suggests that the clinician should choose the treatment sequence based on the skeletal and occlusal conditions of their patients at the start of treatment. **Conclusion:** Early therapy of third skeletal classes with sagittal expansion using a rapid palate expander can be performed earlier or later than posterior-anterior traction with a Delaire mask.

## 1. Introduction

The definition of malocclusion was first introduced by Angle in 1899 and referred to the relationship between the first molars [1]. In this classification, the relative position of the maxilla and mandible was not considered, but the name of the dental malocclusion was associated with the skeletal one even if they did not always coincide. Skeletal class III is defined as a condition of disproportionate forward mandibular growth or deficient maxillary growth. In 1966, Tweed divided the third class into two subtypes: type A, when the mandible is normal in size, and type B, when there is reduced growth of the maxilla or an oversized mandible [2]. Moyers later focused on pseudo-class III, which is a functional anterior sliding condition of the mandible in subjects with retro upper incisors [3].

Sanborn observed that 45.2% of his class III cases had a normal positioned maxilla and an advanced mandible, while maxillary retrusion with a normal positioned mandible was present in about 33% of cases. The remaining 21.8% of cases were divided between normo-position of both bone bases but dental malocclusion or a combination of maxillary narrowing and mandibular protrusion [4].

The term skeletal class III indicates composite of dentoskeletal patterns and has a multifactorial etiology with a prevalent genetic component, but there is also an environmental involvement [5].

A recent meta-analysis reports that the genetic factors most involved in the genesis of the skeletal class III are gene variants at MYO1H (rs10850110), BMP3 (rs1390319), GHR (rs2973015, rs6184, rs2973015), FGF7 (rs372127537), FGF307 (rs593307), and SNAI3 (rs4287555). However, an extremely heterogeneous picture emerges from the study, and the authors conclude that skeletal class III is a polygenic trait substantially modulated by ethnicity [6].

The combination of the use of a face mask and rapid palatal expander (RPE) guarantees greater advancement results of the upper jaw compared to the single use of the mask. In the literature, there are several application protocols of combined therapy, such as alt-RAMEC or Liou, which consist of modified alternate rapid maxillary expansion and constriction. There is limited evidence in the literature demonstrating a better efficacy of these protocols compared to traditional maxillary expansion associated with facial masks [7,8,9,10].

Starting from the assumption that about one-third of patients with skeletal class III malocclusion have a deficit of the upper jaw and supported by studies on the mobilization of the circum-maxillary sutures, we aimed in this study to identify if there was an advantage in performing transverse maxillary orthopedics followed by sagittal advancement or if the reverse sequence allowed for equal or better skeletal results.

## 2. Materials and Methods

In this preliminary study, the cephalometric changes obtained in two groups of skeletal class III patients were compared and treated in the early stages of mixed dentition with a face mask and RPE anchored to the deciduous second molars. The two study groups presented inverted treatment sequences to evaluate whether the different modalities led to statistically significant cephalometric changes for the purpose of the best clinical outcome. The two groups were both made up of 13 patients subdivided into group A, those whose sequence involved the use of extraoral traction first and then the disjunctor, and those with an inverted sequence in group B. Group A had 5 males and 8 females, and in group B, there were 8 males and 5 females, as reported in Table 1.

The same table shows the distribution by skeletal divergence of patients, assuming 32° ± 2° as the normo-divergence interval.

The overall duration of treatment was 1 year and 5 months +/− 6 months for group A and 1 year 3 months +/− 8 months for group B, as reported in Table 2. The minimum age of patients in T0 was 6 years, and the maximum age of patients in T0 was 8 years. It is evident, in T0, the overlap by age of the sample of children treated with RPE and then Delaire facial mask (6 years 11months +/− 10 months) and with Delaire facial mask and RPE (7 years 3 months +/− 5 months).

The criteria for inclusion in the experimental group were the absence of mandibular slipping or centric occlusion–centric relation discrepancy and the presence of at least one of the following characteristics: anterior crossbite, ANB angle equal to or less than 0°, and Wits appraisal equal to or less than 0 mm. All patients were treated consecutively by the same operator using Haas-type rapid palate disjunctor and anterior maxillary traction with Delaire’s mask anchored to splints banded on the second deciduous molars.

All the palatine disjunctors were anchored on bands cemented to the upper deciduous second molars instead of to the first permanent molars, and the extraoral traction coupling splints were built with 1.1 mm. Chrome–cobalt wire, tempered after modeling, with hooks for the rubber bands positioned at the level of the upper deciduous canines. The activation of the quick-release device of the palate took place starting from the same day in which the appliance was cemented, making the patient perform 1/4 to 2/4 of a turn per day and considering the transverse orthopedic phase finished when an overcorrection was obtained relative to the first molars with an almost head-to-head transverse relationship.

The orthopedic traction was instead worn by the little patient for the whole night and four hours during the day. However, daytime collaboration was only achieved by five patients. The activation of the maxillary traction was achieved by using two ½ “14 Oz elastics pulled with a forward and slightly downward direction, angled about 30° with respect to the occlusal plane, and a developed force of about 350 g each.

The active phase of treatment ended when a significant overcorrection in the sagittal plane and class II occlusal relationships was obtained. Sagittal hypercorrection was observed to be maximal on the day of suspension of elastic traction and decreased by about one-third a few weeks later due to recurrence of posterior and downward displacement produced by the chin rest of the mask. For this reason, in group B (RPE + Delaire), the end of the active phase was documented and radiographically recorded on average 7 months after the suspension of the active traction phase, after performing an occlusal balancing and stabilizing the occlusal relationships in “centric relationship”. Even in group A (Delaire + RPE), the end-of-treatment radiograph was performed on average 9 months after blocking the activation of the circuit breaker.

On the patients of both groups, two lateral X-rays of the skull were performed in the usual occlusion position and natural head position (Siemens OP10S) in the following moments: T0 at the start of the treatment and T1 to bone stabilization after the two orthopedic phases.

A cephalometric trace was performed on each X-ray by the same operator, identifying the measurements shown in Table 3.

For each measurement, the mean and standard deviation within the sample were calculated. For each therapeutic interval, the Student’s *t*-test was then calculated for paired data. The two groups were then compared using the statistical significance of *t* for unpaired data. The method error evaluation was calculated using the Dahlberg formula, repeating 12 measurements in five patients one week after the first measurement. An SDE of 0.047 was obtained, which was well below the limit of 0.25.

## 3. Results

The results of the study are shown in Table 4, Table 5 and Table 6.

As shown in Table 4, considering the T1–T0 of Group A (Delaire + RPE), the evaluation of the results obtained in this work allows us to observe how within group A there is a significant improvement in the Witts and Nanda indices following treatment, an index of the differential advancement of the two maxillaries. The skeletal divergence has also changed considerably the inclination of the bispinal plane on the skull base. The convexity index also appears to be statistically significantly improved.

Group B (treated with the palate disjunctor sequence followed by traction with Delaire’s mask) showed a significant improvement in ANB, in AoBo, and AppBpp values and in convexity indicating the clear advancement of the upper jaw following the therapy. On the other hand, no significant increase in skeletal divergence was observed during active treatment. This information is provided in Table 5.

In Table 6, a statistical evaluation of comparison of the two groups in the study shows the values of the two groups, highlighting how in T0 there is a significant difference (*p* < 0.05) in the values of SNA and ANPog. At T1, on the other hand, the two groups were comparable, and no statistically significant difference was highlighted.

The data were analyzed with the software package STATA 16 (College Station, TX, USA).

## 4. Discussion

The treatment of the skeletal class III represents in many cases a challenge for the orthodontist, as the predictability of the treatment is linked to the unpredictable growth of the bone bases, in particular the mandibular one.

The literature agrees that among the malocclusions, the third class is the least widespread [11]. However, this occurs predominantly in the Asian population [12]. The skeletal class III can present as an isolated trait or as a characteristic within pathological or syndromic pictures: in the case of patients with cleft lip and palate, this type of malocclusion is present in 41.7% of the children observed [13].

In a recent study, it was observed that 69.1% of patients with craniosynostosis have a skeletal class III [14]. The resolution of the class III can be obtained, depending on the case, with orthopedic, orthodontic, orthopedic–orthodontic therapy, or orthognathic surgery.

Chin-cup was the first orthopedic therapy for third skeletal classes. The results in the literature are discordant, and some authors have observed negative effects at the joint level [11]. The inability to displace the mandible posteriorly and limit its growth led to the abandonment of the use of the chin-cup replaced by extraoral traction using a facial mask (Delaire or Petit model). This appliance produces forward displacement of maxilla, backward displacement of mandible by clockwise rotation of the mandibular plane, and counterclockwise rotation of the maxillary plane. A meta-analysis states that orthopedic face mask therapy is helpful in resolving short-term skeletal third class [15,16]. Further high-quality, long-term studies are recommended [17].

Pure orthodontic treatment is the ideal solution when the skeletal problem is absent or minimal. It has a dentoalveolar compensation action obtained, generally by means of a lingual inclination of the lower incisors and a proclination of the upper incisors associated or not with the extraction of permanent teeth [18,19]. Mixed treatment, a combination of orthopedic and orthodontic treatment, is still the most clinically widespread type of treatment. A first orthopedic phase, generally carried out in an early phase in deciduous or early mixed dentition, is followed by an orthodontic phase at the end of the exchange.

In some cases, the overgrowth of the mandible or the deficit of the upper jaw make it necessary to perform a combined orthodontic–surgical treatment. Orthognathic surgery can be performed on a single jaw or a bimaxillary intervention [20,21]. The choice of treatment for the skeletal class III resolution must be based on a careful evaluation of the case and an accurate choice of treatment timing.

In the literature, there are many studies with conflicting results. From what emerges in the present analysis the treatment performed with a Delaire mask followed by a RPE (group A) leads to a significant improvement of AoBo and AppBpp and of facial convexity. These results are in agreement with the studies of Kurt [22], Cozza [23], and Krneta [24]. The results differ from the works of Chong [25] and Kapust [26] in that a statistically significant improvement in the ANB angle is not observed.

This is probably because in most of the patients of group A, at the time of the start of treatment with the Delaire mask, the deciduous incisors were present in the arch with the crown of the permanents in a subcortical position precisely in correspondence with point A. Consequently, point A was measured on a convexity rather than a concave area and was, at T0, in a more advanced position than at T1 when, after the eruption of the permanent central incisors, point A is in the center of the concavity supported by the root of the permanent incisors.

In group B, a statistically significant increase of ANB, AoBo, and AppBpp was observed, as reported in most of the studies done on sagittal traction, which have been already mentioned. This could in part be linked to the fact that the use of a transverse expander before traction can result in an anterior movement of point A. Compared to group A, on the other hand, no increase in divergence has been observed either mandibular or palatal.

Post-mandibular rotation was not detected, as was also the case in the Mermingos [27] study. According to the authors, this is because the first permanent molars are free to intercuspate autonomously, as the RPE is anchored to the deciduous molars.

In both groups, no type of dental compensation was observed for the upper incisors or for the lower ones. This is because the moment of the first intervention was, for both study groups, very early. In group A, in which only deciduous teeth are very often present at T0 (65% of subjects), the dentoalveolar incisor compensation, which is associated with the use of the Delaire mask, would lose its importance at the end of active treatment when the improvement of the skeletal relationships would allow the physiological eruption and with normal relationships on the skeletal bases of the permanent incisors. In group B, the very early intervention (7 y, 3 m +/− 5 m), often in the presence of only the upper central incisors, would not allow the establishment of a dentoalveolar compensation because the type of advancement of the upper jaw is quite important, as shown by the remarkable increase in AoBo and AppBpp, which are both statistically significant.

The statistical comparison between the two groups, however, showed that there is no significant difference between the two operative sequences.

In both groups covered by the study, the two operative modalities adopted gave clinical results useful for the treatment of class III malocclusion. Both sequences proved to be equally effective in the results, which are superimposable from a statistical point of view, as the Student’s *t*-test has no significance for any of the cephalometric values considered. The difference in the choice of which is the best operative sequence in the early treatment of the III skeletal classes is therefore purely clinical.

The presence of an anterior cross bite in a very early period, before the eruption of the first molars and permanent incisors, suggests the use of extraoral traction in the first instance. Furthermore, the absence of the first definitive molars in the arch does not allow to correctly quantify the amount of palatal expansion required.

This study has some limitations relating above all to the small size of the sample. Having more patients available to treat would have resulted in more reliable results. Furthermore, the children all come from a single region of Italy: it could be interesting to include in the sample, stratifying it appropriately, even children of other origins.

Furthermore, the evaluations were performed on cephalometric analyses performed on lateral–lateral teleradiographs of the skull: therefore, there is always an uncertainty regarding the precision of the anatomical landmarks.

## 5. Conclusions

The treatment of third skeletal classes at an early age is a widely used therapeutic choice in orthodontics. The use of a rapid palate expander combined with a facial mask is an effective tool for correcting dentoskeletal malocclusion. From what emerges from the present study, the therapeutic sequence does not involve significant differences on the treatment results and must be set by the clinician based on the dentoskeletal conditions of the individual patient at the start of treatment.

## Figures and Tables

**Table 1:** Distribution by age and divergence of the sample groups.

		GROUP A	GROUP B
Sex	Male	38%	62%
	Female	62%	38%
	Hypodivergent	0%	0%
Divergence	Normodivergent	15%	8%
	Hyperdivergent	85%	92%

**Table 2:** Treatment timing.

	T0	T1	T1-T0
GROUP A	6 Y 11 M +/− 10 M	8 Y 4 M +/− 7 M	1 Y 5 M +/− 6 M
GROUP B	7 Y 3 M+/− 5 M	8 Y 7 M +/− 11 M	1 Y 3 M +/− 8 M
T. STUDENT	0.5401	0.24	0.0797
	ns	ns	ns

**Table 3:** Skeletal and dental relationships evaluated in the study.

Sagittal Skeletal Relations	SNA	Position of the Maxilla
	SNB	Position of the mandible
	ANB	Intermaxillary sagittal relation
	SN/GoMe	Skull base/mandibular body relation
	NAPog	Skeletal convexity
	AoBo	Basal relationships on the occlusal plane
	App-Bpp	Basal relationships on the mandibular plane
Vertical skeletal relations	GoGn^SN	Mandibular plane inclination
	SnaSnp^SN	Maxillary plane inclination
	GoGn^SnaSnp	Intermaxillary relationship
Dental	+1^SnaSnp	Upper incisor inclination
	−1^GoGn	Lower incisor inclination

**Table 4:** Study results for the Delaire + rapid palatal expander sequence.

	T0		T1		T1–T0		
	AVERAGE	SD	AVERAGE	SD	AVERAGE	SD	T
**SNA**	81.77	4.92	82.35	3.74	0.58	3.61	**ns**
**SNB**	80.08	4	78.92	3.58	−1.16	3.01	**ns**
**ANB**	1.69	1.55	3.42	1.35	1.73	2.06	**ns**
**AoBo**	−4.23	2.42	−1.73	1.72	2.5	3.11	*
**AppBpp**	2.46	1.94	5.65	1.92	3.19	2.64	*
**PM^SN**	37.85	3.94	38.65	4.92	0.8	2.34	**
**PM^pp**	28.81	3.9	30.88	4.36	2.07	3.45	**ns**
**pp^SN**	9.04	2.35	7.7	3.73	−1.34	3.19	*
**+1PP**	104.2	8.43	114.9	5.54	10.7	9.33	**ns**
**−1PM**	86.8	5.85	88.5	5.44	1.7	6.14	**ns**
**+1−1**	140.78	11.85	125.7	8.68	−15.08	13.04	**ns**
**NAPog**	175.38	3.41	172.8	3.08	−2.58	4.63	**

* p value < 0.05, ** p value < 0.01.

**Table 5:** Study results for the rapid palatal expander + Delaire sequence.

	T0		T1		T1–T0		
	AVERAGE	SD	AVERAGE	SD	AVERAGE	SD	T
**SNA**	78.39	3.06	80.58	3.78	2.19	3.98	**ns**
**SNB**	78.34	2.22	78	3.56	−0.34	3.37	**ns**
**ANB**	0.42	1.99	2.58	2.29	2.16	2.17	**
**AoBo**	−4.92	1.61	−2.96	2.89	1.96	1.9	**
**AppBpp**	1.96	2.31	4.31	3.89	2.35	2.79	*
**PM^SN**	38.5	2.97	38.65	5.38	0.15	4.16	**ns**
**PM^pp**	10.92	4.5	9.96	2.77	−0.96	4.4	**ns**
**pp^SN**	26.8	6.74	28.61	5.7	1.81	4.66	**ns**
**+1PP**	110.96	10.77	115.46	6.18	4.5	10.02	**ns**
**−1PM**	84.73	7.1	87.73	5.85	3	5.41	**ns**
**+1−1**	135.04	16.13	128	8.51	−7.04	12.55	**ns**
**NAPog**	179.3	4.92	175.35	5.87	−3.95	4.61	**

* p value < 0.05, ** p value < 0.01.

**Table 6:** Results of the comparison between the two groups covered by the study and statistical significance.

	T0		T1		T1–T0	
**SNA**	0.048	*	0.242	**ns**	0.289	**ns**
**SNB**	0.189	**ns**	0.516	**ns**	0.526	**ns**
**ANB**	0.084	**ns**	0.265	**ns**	0.616	**ns**
**AoBo**	0.4	**ns**	0.203	**ns**	0.601	**ns**
**AppBpp**	0.556	**ns**	0.279	**ns**	0.435	**ns**
**PM^SN**	0.637	**ns**	0.998	**ns**	0.627	**ns**
**PM^pp**	0.181	**ns**	0.123	**ns**	0.063	**ns**
**pp^SN**	0.197	**ns**	0.103	**ns**	0.063	**ns**
**+1PP**	0.088	**ns**	0.83	**ns**	0.112	**ns**
**−1PM**	0.424	**ns**	0.732	**ns**	0.57	**ns**
**+1−1**	0.313	**ns**	0.5	**ns**	0.122	**ns**
**NAPog**	0.028	*	0.178	**ns**	0.465	**ns**

* p value < 0.05.
